# Stricture of the Urethra1Read at the thirtieth annual meeting of the Medical Association of Central New York at Buffalo, October 19, 1897.

**Published:** 1898-07

**Authors:** J. Henry Dowd

**Affiliations:** Buffalo, N. Y.; Genito-urinary surgeon Buffalo Hospital Sisters of Charity, etc.


### STRICTURE OF THE URETHRA.[1]

By J. HENRY DOWD, M. D., Buffalo, N. Y.
Genito-urinary surgeon Buffalo Hospital Sisters of Charity, etc.

I TRUST you will pardon me if I say at once that I shall not burden you with that which is available to all and can be read and studied at a more opportune time than the present, when there is before this society subjects of vaster importance; but I shall hurriedly say a few words of the cause, pathology, symptoms and of the more important part, the treatment. Urethral stricture may be congenital or acquired. I cannot give statistics only to say that from the examination of the urethra of at least 200 males I have never but twice found a well-formed stricture occurring in patients who could give no history of injury and had never had a gonorrhea until four or five weeks before they were sent to me.

The contractions were $2\frac{1}{2}$ and $4\frac{1}{2}$ inches from the meatus, the former one catching a 24 and the latter a 22 bulb. In front and behind, the canal measured 30 F. The acquired variety, as you well know, may be due to injury, let it be external violence or the impaction of a foreign body in the urethra, instrumentation or specific inflammatory conditions within—the latter being the cause of 98 per cent. of all strictures. The acquired variety we may divide into inflammatory, traumatic, spasmodic and organic. I think the first should be dropped. The fact is not disputed that during an acute attack of gonorrhea the caliber of the canal is lessened in a marked degree, but as the inflammation subsides the normal is again reached, and provided the case has been properly treated no localised spots remain which can become the foothold for a commencing cicatrix. On the other hand, chancre or chancroid will generally be followed by contraction, owing to the depth of the tissue involved. Looking at strictures as something of permanency until relieved by operation, I am in doubt whether the name spasmodic should not be changed to urethro-muscular spasm. The fact cannot be denied that on passing a sound or other instrument for the first time into the urethra of a

1 Read at the thirtieth annual meeting of the Medical Association of Central New York at Buffalo, October 19, 1897.

highly nervous person it becomes suddenly stopped, but on waiting a few minutes it readily enters the posterior urethra. Retention will also take place from spasm of the muscles, due to reflex causes, or as every one has experienced from operations around the rectum or perineum; still I am inclined to think the last condition more often due to inhibition or blunting of the nerves governing the urination sensation.

Traumatic injuries to the perineum and penis are fruitful causes of stricture, and let me say right here these strictures from their incipiency are best dealt with by the knife; in other words, cutting them seems to be the only remedy through which we can hope for results. By far the most common injury is due, as the laymen say, to "breaking a chordee," or by forcibly straightening a curved penis during erection, the mucous membrane being torn with a resulting cicatrix.

*Organic.*—Under this head we find the cause of at least 98 per cent. of urethral strictures and the all-important cause is gonorrhea. I have never seen a stricture due to masturbation; still, on the other hand, I have seen and treated a case where retention of urine lasted for four days, due to the injection of a strong solution of bicloride, yet no stricture followed. The two latter classes can be divided into large and small and these again into linear, anular and tortuous, which may be from one to two or even three inches long. Further, a division can be made into irritable, which always bleed freely, and resilient, which on being stretched at once assume their former size. The symptoms of stricture as put down in the text-books are very misleading; suffice it to say, however, that the caliber of the canal must be very much lessened; in fact, a stricture to conform to the symptoms generally found in text-books must be on an average of from seven to ten years old. There are important symptoms which arise that should lead us to an exploration of the urethra, thus diagnosticating contractions before the tissue takes on a cartilaginous character. A clear urine containing shreds consisting of pus cells, held together by mucus, should be just cause for examination of the canal, where in all probability a contraction will be found, behind which is a chronic inflammation, furnishing the above named shreds. Constant burning of the glans in the absence of any inflammatory manifestation, perineal pains, pains shooting down the inside of the legs, pain in the back or soles of the feet, repeated glandular herpetic eruptions, headache along the occiput and vertex, should always cause the urethra to be examined for contractions. I have on my history book and reported at the academy of medicine a case of pronounced dyspepsia which disappeared entirely on dilatation of his stricture. For other symptoms I shall have to refer you to any good book on genito-urinary surgery. The location of stricture depends mostly on the vascular and dependent portion of the canal, and this we find at or near the bulbo-membranous junction, where 67 per cent. of all strictures are located, the other 33 per cent. being one inch behind to two and a-half inches in front of the peno-scrotal angle.

Before speaking of the detection of stricture let me say a word as to large and small calibers, for on this point authors disagree. Some claim that when a No. 18 F bulb is caught it indicates a small caliber. Hayden in his book gives no place to them, but speaks of chronic localised inflammations, this probably conveying his opinion of such conditions. I am inclined to the opinion that, using the Otis measurement of the flaccid organ as a standard, a bulbous bougie that catches indicates that stricture exists; if none is found and still symptoms continue the canal should be examined by the electro-endoscope. If the mucous membrane is normal it will as the tube is withdrawn close around the end as though tied by a string. On the other hand, when localised inflammation, especially involving the submucous tissue, there will be a sort of funnel formed, simply indicating that due to the underlying inflammatory processes the membrane cannot fall together as when normal. At these spots generally will be found localised foci of inflammation—possibly granulations. Reginald Harrison says "nature to prevent a leakage of urine throws out plastic material as a pavement and this in time changes into tissue having a tendency to contract, thus stricture is formed." These funnel-shaped spots I will call stricture of large caliber and which if left alone will in time form true stricture, being able of detection with a bulbous bougie. For the detection of stricture it is advisable to use a bulb. The Otis urethrotome at times will deceive you.

A word or two as to the remote damage caused by urethral stricture and especially so if of long standing and very small caliber. Cystitis in a chronic form usually develops from along continued obstruction to the passage of urine, and when well-seated is a very difficult thing to cure. Ureteritis and pyelitis are more common results of stricture than has been thought. To have pain in the back before pyelitis is thought of is as absurd as to think that a patient with chronic nephritis must have backache before we think of diagnosticating Bright's disease. The disease pyelitis when ascending, I am almost safe in saying, is incurable in the male; in the female, where it is possible to wash out the pelvis of the kidney with astringents, we may hope for results. Ascending nephritis or surgical kidney is beyond the aid of drugs, excepting in the acute stage. In this, as in the former, we must remove the obstruction and trust in God for the ureter not to get plugged with pus, resulting in pyonephrosis, where the only remedy is nephrectomy. Bright's disease may also follow a long continued obstruction to the free flow of urine. In tight strictures there is always more or less prostatitis and the seminal vesicles are at times enlarged and swollen.

*Treatment.*—Should a person ask me, as I frequently am asked, is stricture curable? I should say yes, by cutting. In innumerable canals examined by me by aid of the electric light, I have invariably found that where cutting has been done, with an interval of years without the passage of an instrument no contraction is noticeable. But is it advisable to cut every case? Decidedly no. The risk of life is 2 per cent., not always by hemorrhage or sepsis, but frcm shock. Then permanent curvatures will result and impotence will develop, from which there seems to be no appeal. I shall again refer to the old saying, an ounce of prevention is worth a pound of cure. Prevent stricture and you will have none to cut or dilate. Leaving out of the question traumatic, which, of course, cannot be prevented, except those due to breaking a chordee, the latter not occurring if a little explanation is given the patient, we find that most urethral strictures are due to one cause—gonorrhea, and when this is treated as it should be, no complications should remain behind. After a protracted inflammation of the urethra, when all discharge has stopped and the urine is clean, the canal should be examined by electric light. This will reveal to you any localised spots of inflammation, erosions, granular spots or funnel-shaped depressions, conditions that to me are the danger signals that time will change the lesion into that of stricture.

It is at this time that we may prevent these formations if a vigorous course of treatment is carried out by sounds, followed by a very weak solution of silver nitrate, thrown over the entire mucous membrane. Cases coming to us where stricture already exists call for surgical judgment. Shall we dilate or must we cut is the all-important question? I think we should make a fair decision at the first visit, if we use care in the examination. If conditions are found, such as I shall later describe, by all odds the safest and quickest is to cut. With a history from three to five years old, let them be anular, linear or tortuous, good results will follow gradual dilatation. In uncomplicated strictures, that is where the urine shows no great amount of trouble, either in front or behind the contraction, and where the canal is not inflamed or congested, evidenced by the amount of pus or mucus in the test urine or discharge, no instrument will give the results that can be obtained with the Oberlander dilator, and this with a minimum amount of pain to the patient. It is a well-known fact that the dilatation of the meatus gives more pain than the stricture. Should this be of smaller caliber than the 26 it is well to divide it to 30 F, but not to 35 or even 40 as we see occasionally. As the urethra is not a tube of uniform caliber, but normally 30 to 35 at the bulb and this the seat of 67 per cent. of strictures, you can see by looking at the instrument that it is built to conform with a normal urethra; it therefore dilates the same accordingly. Although we see strictures through which at first it is impossible to pass a filiform, the lumen of the urethra is never completely obliterated: there is always an opening let it be but the size of a hair. The chief cause of failure to pass a filiform is due to the extreme sensitiveness of the contraction and its surrounding tissue, spasm occurring as soon as an instrument touches it. Cocaine will not always facilitate a passage, but deep narcosis has never failed me. Where there has been retention for hours, say twelve to fifteen, and particularly if the stricture is of the old fibrous variety, unless the filiform can be easily passed, suprapubic puncture should be resorted to at once and the stricture looked after later.

Rarely will a stricture be found through which a filiform cannot be passed; when one is in, pass more if possible; if not, leave the first in situ for twenty-four hours and the urine will trickle by it. It is usually found that at the end of twenty-four hours that a 4 or 6 F can be passed easily; if not, three or four filiforms can be crowded by one at a time. Following the instructions given in any text-book, except in a few cases, the normal caliber should be reached in four to six weeks. Never use a steel instrument in the urethra until 18 or 20 F is reached, when the Oberlander is the instrument that will give the best results. A word about Ford's electroliser. Personally I have had no experience, but I have seen Dr. Ford use it. From the amount of blood and the opinion of eminent men who were present also, and have given the subject some little study, I think it causes more damage than already exists. For some time I have had rules as to cutting and which has served me admirably.

1. Irritable.—Those strictures that bleed every time they are touched and are often followed by chills and fever. Cutting here, let it be external or internal, will give good results and if the patient always has a chill give him one and end them at once.

2. Resilient or rubber strictures, as I speak of them to the layman. These should always be cut. For some reason, no one seems to know why, the tissue is very elastic, contracting to its former size almost as soon as the sound has been withdrawn.

3. Tough, fibrous cartilaginous bands, which the sound seems not to be able to effect, and with much force the urethra is liable to be torn either antero or posterior to the contraction.

4. Under this head can be included impassable or strictures where after a good trial it has been found impossible to pass a filiform bougie. Strictures where rupture of the urethra has taken place, or behind which urinary abscesses have formed; in fact, in the last two conditions there is no alternative. Any contraction three quarters of an inch from the meatus and possibly I might include those with fistula behind. A closure of these can take place, still I think the end sought can be more quickly attained if we restore the canal to its normal caliber at once.

For the technique of internal or external urethrotomy I can only refer you again to any good book on genito-urinary surgery, still a few points may not be amiss. Thorough antisepsis must be carried out. The hands of the operator, instruments, urethra, perineum and glans, also the lubricant, must receive the closest attention as to cleanliness. It is the teaching of some and also their practice in doing internal urethrotomy to cut the whole urethra from behind the last contraction to the meatus. Nothing in my opinion can be more absurd. If anyone can give me a good excuse why healthy mucous membrane should be cut for from four to five inches simply to divide one or even two or three contractions on an average of not over one-eighth in width, I may be converted into dividing it throughout, but until then would advise cutting, say one half inch behind and one half inch in front of the stricture; this will divide all contracting tissue and localised inflammation. In doing urethrotomy, especially to the beginner, it is often a fine point to determine when to do an external and when an internal operation. As I have said before, 67 per cent. occur at or near the bulbo-membranous junction; that is, in the neighborhood of one to two inches in front of the compressor muscle, and these should always be cut externally. I think it is safe to say that a rule that can be followed would be to pass a bulb or other instrument that can be easily felt from the outside, and with the tip of the same resting in the perineum and about one and a half inches in front of the anterior border of the anus.

Any stricture above this point can be cut internally and below it should be divided through the perineum. In external operations the perineal tube in the bladder is an absolute necessity, removing it about the end of the forty-eighth hour if there is not much pus; if there is, it can be left longer, say four or five days, and through it the bladder can be washed three or four times a day. Either after internal or external operations, a practice I have long carried out is to pass a full-sized sound every fifth day until the cut surfaces show no tendency to recontraction, then I think it is safe to say the stricture is cured. The operation of Cook or Wheelhouse's external urethrotomy without a guide is at times the most difficult operation in surgery. In all cases where the guide cannot be passed I am in favor of the Wheelhouse minus the hook. To puncture the urethra at the apex of the prostate is an easy thing on paper, but a most difficult thing to do. In a case where this operation may be called for, viz., the perineum filled with fistulæ, it is wisest, in my opinion, to make a suprapubic cystotomy, pass a grooved staff through the bladder and prostatic urethra and up to the stricture and then cut upon it. Should it be impossible to find the opening by this procedure another director can be passed through the penis and the urethra opened anterior to the stricture. Now, with an open canal in front and behind the obstruction it is an easy matter to divide it from without inward. A thought which almost escaped me and which is of the utmost importance, never use too much force in overriding a filiform bougie by a tunneled instrument and especially when an operation is not to be done at once. In doing so you are liable to cut the whalebone instrument in two and have to operate at once, and externally at that, to find the missing half. Salol or boracic acid in good doses is of value and should be given for from three to four days before operating for stricture. I always irrigate a urethra with a half per cent. formaldehyde solution before passing a sound.

223 Franklin Street.

---

A good cough mixture, in use at Roosevelt hospital, New York:

| | |
|---|---|
| Codein. . . . . . . . . . . . . . . . . . . . . | gr. iv. |
| Acid. hydrocyanic. dilut. . . . . . . . . . . . . . | gr. xv. |
| Ammonii chlorid. . . . . . . . . . . . . . . . . | gr. xl. |
| Syrup. pruni virginianæ . . . . . . . . . . | q. s. ad. f ℥ ij. |

M. Sig.—A teaspoonful every three or four hours.

